# Antimicrobial effects of syndiotactic polypeptides

**DOI:** 10.1038/s41598-021-81394-2

**Published:** 2021-01-19

**Authors:** Prakash Kishore Hazam, Chimanjita Phukan, R. Akhil, Anjali Singh, Vibin Ramakrishnan

**Affiliations:** 1 Molecular Informatics and Design Laboratory, Biosciences and Bioengineering, Indian Institute of Technology Guwahati, Guwahati, Assam 781039 India; 2 Department of Pharmacology and Toxicology, National Institute of Pharmaceutical Education and Research Guwahati, Guwahati, Assam 781125 India; 3 Guwahati Medical College Hospital, Bhangagarh, Guwahati, Assam 781032 India

**Keywords:** Biophysics, Computational biology and bioinformatics, Drug discovery

## Abstract

We present design and antibacterial studies of stereochemically diversified antimicrobial peptides against multidrug-resistant bacterial pathogens. Syndiotactic polypeptides are polymers of alternating L and D amino acids with LDLD or DLDL backbone stereochemical sequence, which can form stable gramicidin like helical conformations. We designed, synthesized and characterized eight model molecular systems with varied electrostatic fingerprints, modulated through calibrated sequence positioning. Six out of eight model systems showed very impressive antimicrobial activity against three difficult to treat bacterial species, Gentamicin resistant MRSA, *E. coli* and Mycobacterium. More importantly, the designed LDLD peptides were equally potent in serum, an important drawback of poly L peptide sequences due to enzyme mediated degradation and ion sensitivity. Further, we tested the activity of the designed peptides against drug-resistant clinical isolates of *Staphylococcus aureus* and *Escherichia coli*. Molecular dynamics simulation studies suggest formation of an assembly of individual peptides, preceding the membrane interaction and deformation. The activity estimates are comparable with the available peptide based antimicrobials, and are also highly specific and less toxic as per standard estimates. Incorporation of D amino-acids can significantly expand the peptide design space, which can in turn manifest in future biomaterial designs, especially antimicrobials.

## Introduction

Antibiotic resistance is now a major concern, and discovery of new drug candidates is a growing clinical need^1^. A variety of resistant species^2^ like MDR (Multi Drug Resistant), XDR (Extensively Drug Resistant) and PDR (Pan Drug Resistant) have evolved, but discovery pipeline has already shown signs of decline^3^. Renewed interest in administering short peptides like Polymyxin B1 (PMB1), as ‘last resort’ therapy^4,5^ which was once restricted to only topical use due to its adverse side effects on the renal and nervous system, is a pointer to the acute shortage of new lead candidates in industry^6^. Generally, peptides are ideal candidates owing to their minimal side effects, high tolerability and specificity, which would help them to successfully comply with the prescribed safety standards, set for clinical trials^7^. Low bio-availability and ion sensitivity are some of the major drawbacks that limit their utility. The reduced bioavailability and ion sensitivity of the peptide molecule can be attributed to limited absorption, metabolic factors like pH, and enzyme mediated degradation^8–11^. These enzymes are ubiquitous in different levels of cellular organizations, in body fluid and extracellular spaces^12^. Usage of D-amino acids and cyclization of peptides has been reported to resist enzymatic degradation and ion sensitivity to a significant extent, which would probably result in better activity in biological fluids^13–15^. The choice of opting peptide based antibacterial agents as a case study for stereo-chemically engineered peptide, pivot around this acute necessity in supplementing new therapeutic options and transforming the option of using peptide drugs to a more potent and effective solution.

Protein molecules are polymers of amino-acids. The spatial orientation of amino acids in natural proteins are exclusively left handed or L-chiral^16^. In polymer chemistry, ‘tacticity’ describes the arrangement of adjacent chiral centers within a given polymeric molecule. A polymer can be isotactic, syndiotactic or hetero-tactic, based on their spatial arrangement. An isotactic polypeptide sequence is characterized by amino acids having L- or D-stereochemistry only. Syndiotactic sequence is defined as having stereo-regular arrangement with alternate L- and D-amino acids or vice versa in succession (LDLD or DLDL). Hetero-tactic polymer has a random distribution of L and D-amino acids in its sequence^17^. Hypothetically, if we add one more variable as ‘stereochemistry of amino-acids’ in the backbone, number of stereo-isomeric diversity increases exponentially from 1^n^ to 2^n^. The entire protein universe, nonetheless, is made of only one of them (poly L-chiral or isotactic), which means (2^n^ − 1) is yet to be explored, and experiments in this direction may have a huge impact in biomaterials research^18,19^.

Chiral engineering of peptide backbone was first utilized by Ghadiri and coworkers. They have designed nano-level molecular assemblies with alternating L and D amino-acids adopting a ring structure, stacked up forming a hollow tube^20^. Molecular constructs based on peptides with adaptive modulation against various membrane types and target functions have been employed in the design of antimicrobial agents, cell penetrating domains and tumor homing molecular devices^21–23^. From this experience, we formulated the following objectives for this investigation: (1) to explore the possibility of exceeding the structural and sequential space of natural proteins by designing ‘fully de novo’ functional units, with a series of peptides, having exceedingly good antimicrobial property as a case study; (2) to design various combinations of electrostatic zones with varying degrees of polarity on a stable syndiotactic backbone, such that, we may be able to deduce specific electrostatic fingerprints, optimized against Gram-positive, Gram-negative or Mycobacterium species and (3) to design peptide molecular systems that can resist enzymatic degradation in biological fluids by evaluating the antimicrobial activity against three different and ‘difficult to treat’ bacterial species including multi-drug resistant clinical isolates.

## Materials and methods

### Design and electrostatic profiling

The design of peptide molecules was performed with the help of a modified version of Ribosome software gifted by Rose laboratory^24^. We used ‘Delphi’ software to compute the electrostatic potential from Barry Honig of Columbia University (http://compbio.clemson.edu/sapp/delphi_webserver/). The electrostatic potential was calculated at the chromophoric centers of the respective side chains^21^. Three-dimensional graph of chromophoric centers of each amino-acid was plotted using MATLAB software (licensed to IITG), showing distinct electrostatic potential signature.

### Molecular dynamics simulations

Molecular dynamics simulations were performed for 200 ns to investigate the translocation mechanism of the peptides AS03, AS05, AS06 and AS08 across bacterial model membrane composed of phospholipids, Palmitoyl-oleoyl-phosphatidylcholine (POPC) and 1-palmitoyl-2-oleoyl-sn-glycero-3-phosphoglycerol (POPG) in the ratio of 3:1^25^. POPC and POPG lipids^26^ were obtained from Mem-Builder version 2.0^27^. The simulations were performed using GROMOS 53 A6 force field in the GROMACS 5.0.4 package, an open source molecular simulation program suite)^28^. Four copies of the peptides were aligned, 1 nm above the lipid bilayer. The topology files of the lipid bilayer and the peptides were concatenated to obtain a single file. The lipid parameters were obtained from berger et al.^28^ and the peptide parameters were from GROMOS 53a6 force field^28^. Open source tool VMD is used for visulatisation (https://www.ks.uiuc.edu/Research/vmd/).

Simple point charge water was added to peptide lipid system after enclosing it in a triclinic box with a spacing of 1.0 nm from the box edge, to mimic the hydrated cellular lipid environment. Overall charge of the system was brought to neutral by adding counter ions, thus increasing the stability of the simulation. Energy minimization was carried out using the steepest descent method, until a tolerance of 1000 kJ mol^−1^ nm^−1^ was reached. The temperature was set at 303 K under NPT conditions at a pressure of 1 bar. A pressure coupling constant was set using Parrinello–Rahman barostat algorithm, and isothermal compressibility values were set at 4.5 × 10^−5^ bar^−1^ for simulations in explicit water. Throughout the experiment, the trajectory files were analyzed using VMD^29^ and PyMol^30^. The membrane thickness was calculated using GridMAT-MD^31^ program for comparative analysis of the simulated membrane. The thickness between the outer and the inner leaflet was measured and plotted using gnuplot^32^.

### Peptide synthesis and purification

The peptide synthesis was performed on a solid support (Rink amide resin) using standard N-9-fluorophenylmethoxy-carbonyl (F-moc) protocol^21^. 20% piperidine was used for the removal of N-terminal F-moc group, followed by washing to attain neutral pH. A three-fold excess of amino acid, *N*,*N*,*N*,*N*-tetramethyl-*O*-(1*H*-benzotriazol-1-yl) uroniumhexafluorophosphate (HBTU), 1-Hydroxybenzotriazole (HOBt), and six-fold excess of *N*,*N*-diisopropylethylamine (DIPEA) was used for coupling in two separate cycles of 60 min followed by 30 min for every amino acid. Post synthesis, a cleavage cocktail (m-Cresol: Thioanisole: EDT: TFA::2:2:1:20) was used to separate peptide from resin. Reverse phase HPLC (Shimadzu, LC 20AD, C-18 (Merck) ) run, 10% to 100% (gradient) acetonitrile containing 0.1% Trifluoro acetic acid (TFA) at 0.5 mL/min was used to separate peptides (≥ 95%) at 210 nm. The peptides were characterized by MALDI (Bruker, Autoflex Speed) mass spectrometry. All the peptides have amidated C-terminal due to the use of rink amide resin for peptide synthesis.

### Antimicrobial and serum sensitive assay

The antibacterial activity was performed using micro-dilution method as per Clinical and Laboratory Standards Institute (CLSI). Gentamicin resistant MRSA (ATCC 33592), *Mycobacterium smegmatis* (ATCC607) were procured from ATCC, *Escherichia coli* MG1655 has been sourced fromChaudhary laboratory, BSBE, IIT Guwahati. The clinical isolates was provided by Dr. Chimanjita Phukan (Guwahati Medical College Hospital, Bhangagarh, Guwahati, Assam). The media broth was procured from Himedia Laboratories. Overnight culture of Gentamicin resistant MRSA (ATCC 33592), *Escherichia coli* MG1655, clinical isolates [(*S. aureus* (drug resistant) and *E. coli* (multidrug resistant) supplementary table 2] were diluted to 10^6^ CFU/mL in Nutrient Broth (NB). 100 µL of broth containing 50 µL of bacterial suspension and test peptides at various concentrations were incubated for 12 to 16 h at 37 °C^33^. Similarly, *Mycobacterium smegmatis* (ATCC607) was diluted to 10^6^ CFU/mL in M7H9 media. 50 µL of bacterial suspension was treated with 50 µL peptides at various concentrations. The peptide bacterial suspension was incubated for 72 h at 37 °C. The antimicrobial potencies of the peptides were evaluated at 600 nm by a comparative assessment with untreated bacterial suspension (negative control). The Minimal Inhibitory Concentration (MIC) was reported at the lowest concentration, where no growth was observed, as per standard protocols. The serum sensitivity experiment was similar to antibacterial activity. In this experiment, 50% serum containing media was used, instead of media alone^15^. The experiment with human serum was performed as per IHEC (Institute Human Ethics Committee) guidelines, at IIT Guwahati hospital, from healthy adult donors. All the protocols were approved by the IHEC, and performed, with the informed consent and written approval from the subjects.

### Field emission scanning electron microscopy (FE-SEM)

Post incubation, bacterial suspension was treated with glutaraldehyde (4%) for 30 min. The bacterial cells were washed (1500 × g, 5 min) with buffer (10 mM phosphate buffer, pH 7.4) and placed over glass slide. A gradient wash with ethanol (30–100%) was performed, followed by gold coating, before imaging^22^.

### Hemolytic assay

5 mL of fresh human blood was mixed with ethylene diamine tetra acetic acid disodium dihydrate salt (EDTA) at a concentration of 2 mg/mL. The Red Blood Cells (RBCs) were re-suspended and washed (800 × g, 5 min) with buffer (5 mM HEPES buffer saline, pH 7.4). 50 µL of 10% cell suspension was mixed with 50 µL peptide at a value close to MIC concentration in most cases (8 µM), followed by incubation at 37 °C for 2 h. The hemolytic potential of peptides was evaluated by comparative estimation of peptide treated, positive control (1% triton x) and negative control (buffer) supernatant at 540 nm^22^. The entire experiment using human blood, has been performed as per IHEC (Institute Human Ethics Committee) guidelines, at IIT Guwahati hospital, from healthy adult donors. All the protocols adopted were approved by IHEC (Institute Human Ethics Committee), and performed, with their informed consent and written approval from the subjects.

## Results

In this project, we have designed eight model systems, out of which seven are having syndiotactic backbone with LDLD stereochemistry and the remaining one (AS07) is an LLDD peptide (Table 1). Traditionally peptide based antimicrobials have two main issues, which limit their potential utility as therapeutic solutions. First is the uncertainty of the designed sequence forming prescribed structure while encountering the membrane and second is the proteolytic stability of the peptides while administering intravenously^15^. Different mechanisms were proposed for membrane lysis of bacterial cell, but the interesting fact is that, the membrane composition is different in different bacterial species. Few common features such as cationicity, amphipathicity, length, secondary structure etc. tend to get repeated in the design of AMPs^34^. But the chances of having the sequences retaining its designed structure while encountering the membrane is debatable. Through this series of experiments, we present eight peptide molecules, which can possibly address the above limitations. The peptides were synthesized and characterized for their antimicrobial activity, toxicity and serum sensitivity using standard analytical techniques. Molecular dynamics simulations provide critical insights about the membranolytic activity of the designed peptides, and possible direction for future designs.

**Table 1:** Peptide code, sequence, molecular weight and their respective MIC values against *Escherichia coli*, Gentamicin resistant MRSA and *Mycobacterium smegmatis* in the presence and absence of serum.

Peptide code	Sequence	Molwt (Da)	*E. coli*		Gentamicin resistant MRSA		*M.smegmatis*		Percent Hemolysis
			Medium	50% serum	Medium	50% serum	Medium	50% serum	
AS01	RkRwWvVkRkWwV-NH_2_	1911.126	3.13	6.25	6.25	6.25	6.25	6.25	7.24
AS02	RkRwLwLkRkWlW-NH_2_	1953.173	6.25	3.13	3.13	6.25	3.13	3.13	0
AS03	KkKwWvKkKwWvV-NH_2_	1827.108	6.25	3.13	3.13	6.25	6.25	6.25	0
AS04	WwKkKvWvKkKwW-NH_2_	1914.119	3.13	6.25	1.6	1.6	6.25	6.25	0.25
AS06	KkKvVvKkKvVwK-NH_2_	1596.086	25	12.5	50	50	25	25	0
AS08	WlWlWlWvKkAkAkK-NH_2_	1982.203	6.25	6.25	6.25	3.13	6.25	6.25	1.46

The MIC values are expressed in micro-molar (µM) units. The mammalian cytotoxicity potential of the peptides is shown as percent hemolysis. Small letters denote D amino acids within the peptide sequences.

### Design, synthesis and characterization

**Figure 1:**
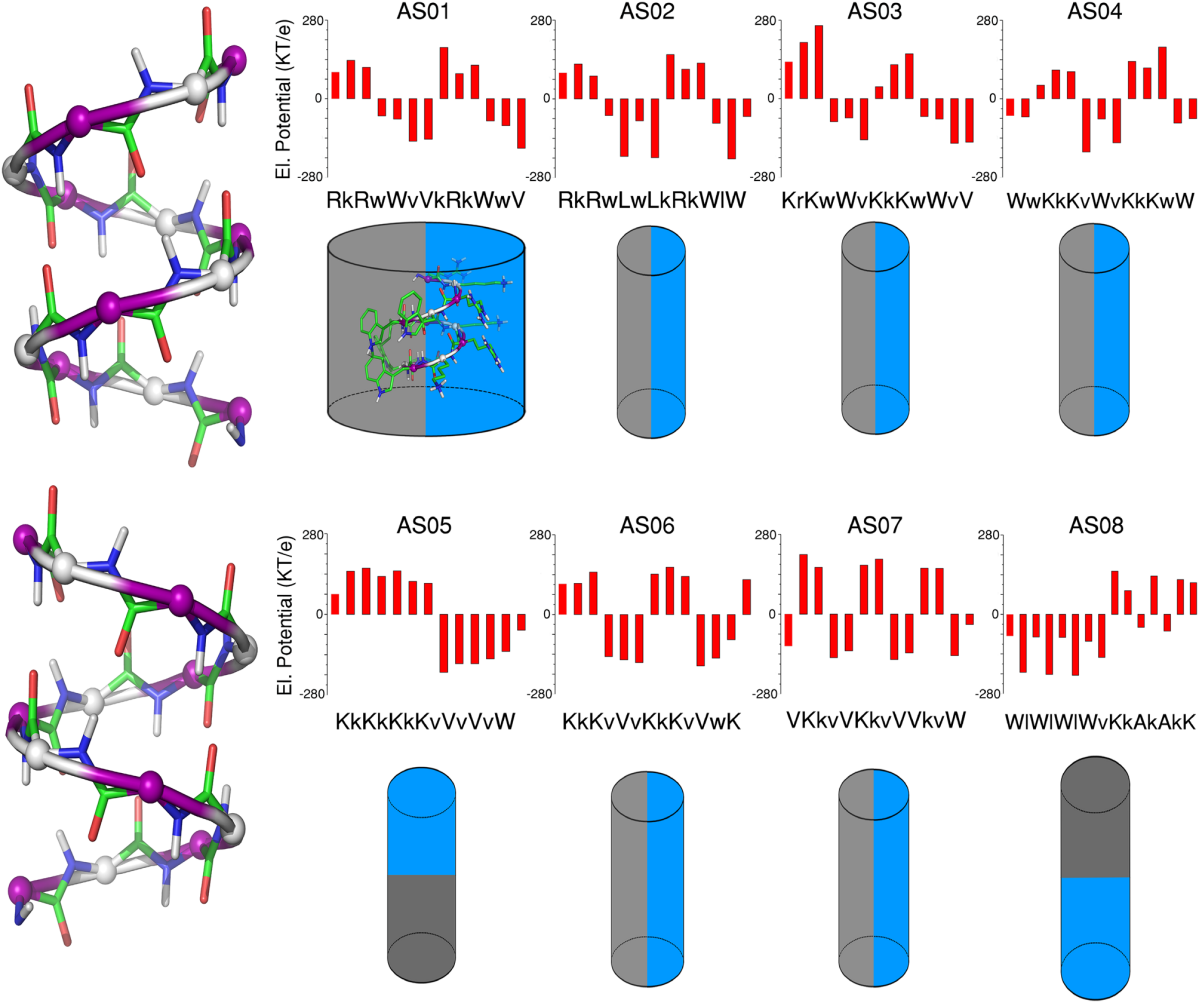
Cylindrical cartoon representation of backbone structure and electrostatic potential of peptide series (AS01–AS08). The electrostatic potential is expressed in KT/e unit. The blue color shows the sequences with cationic side-chains, whereas, the grey color indicates non-polar (hydrophobic) side-chains. Small letter in the sequence represents D-enantiomers of respective amino acids. All the cylinders are having N terminus at the top and C-terminus at the bottom. Right handed and left handed syndiotactic helical molecules with alternating L,D chain stereochemistry is shown as ball and stick diagram. Short range electrostatics of gramicidin beta helix is mutually complementary, with NH and CO bonds with opposite polarity in close proximity. This will improve the overall stability of the molecule, helping to retain the structure and designed amphipathicity while interacting with the membrane. Images of cylinders are made using MS powerpoint, Peptide illustrations using Ribosome (gifted from G D Rose Lab) and Pymol (https://pymol.org/2/).

All the eight peptides designed were amphipathic in nature (Fig. 1). However, the electrostatic fingerprint and amino acid composition were varied in each model system (Supplementary Fig. 1). The peptides AS01–AS04, AS06 and AS07 had longitudinal division of hydrophobic (grey) and cationic (blue) zones, whereas AS05 and AS08 were designed to have hydrophobic and cationic zones in vertical divisions (Fig. 1). Peptides AS05 and AS07 has been excluded from antibacterial assays, because of not having the desired levels of purity.

### In-vitro antimicrobial activity and hemolytic activity of peptides

**Figure 2:**
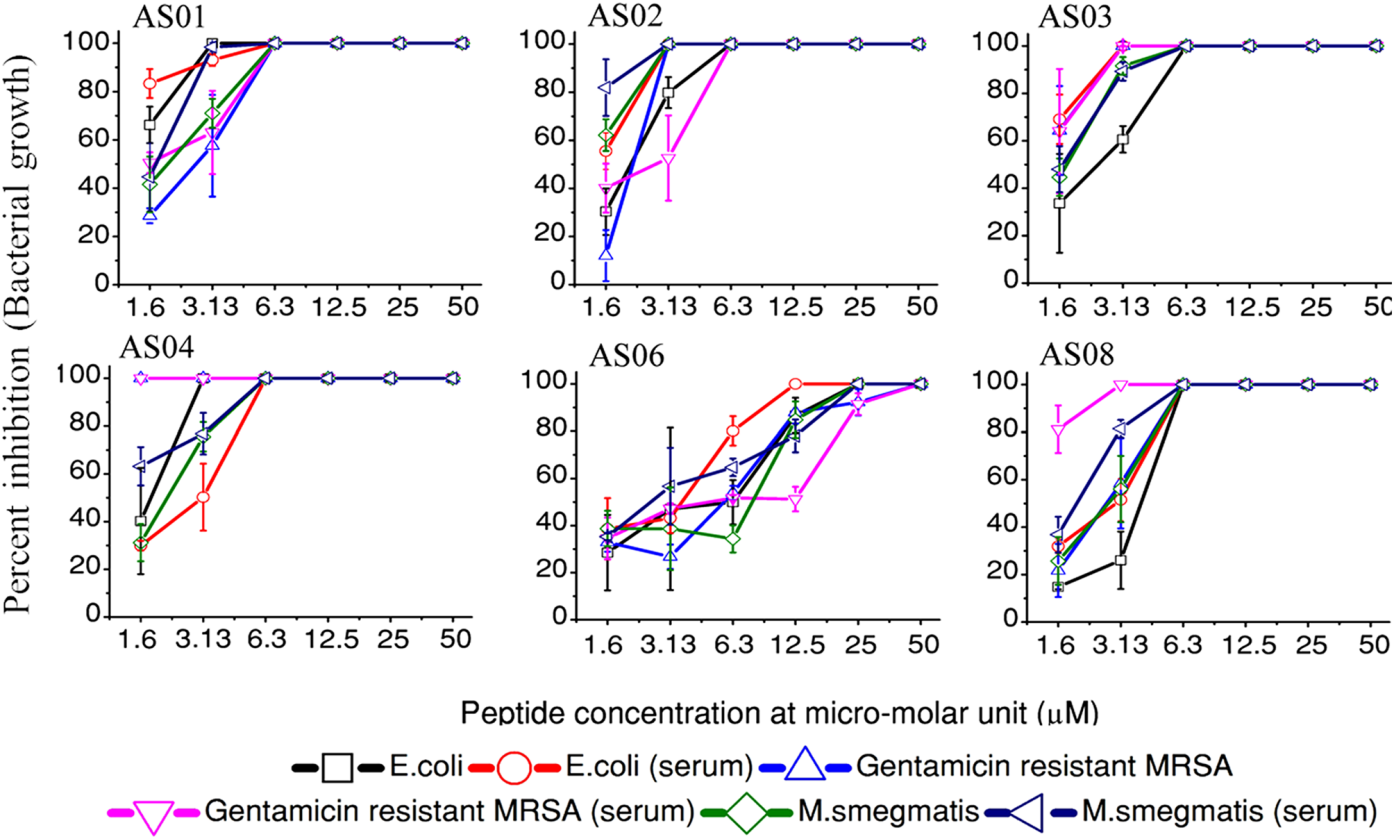
Antimicrobial activity of the designed peptides (AS01–AS04, AS06 and AS08) against *E. coli* MG1655, Gentamicin resistant MRSA ATCC 33592 and *M. smegmatis* ATCC 607 in media and serum containing media (50%). The error bars represent standard. Antimicrobial activity pattern of the designed peptides are distinctly different against different bacterial cell types. This verifies the core idea of how different electrostatics signatures manifested through amino acid sequence on a backbone can act differently against different membranes. However, AS06 was found to be not as effective as other peptides against 3 bacterial species tested.

All the six model peptides systems are showing significant antimicrobial activity. The peptides were designed with different electrostatic potential zones, fixed by its LDLD chain stereochemistry, and manifested through its cationic and hydrophobic side chains. Understandably, Gram-positive (*S. aureus* ATCC 33592), Gram-negative (*E. coli* MG1655) and acid fast (*Mycobacterium smegmatis* ATCC 607) species are different in their morphology and charge distribution of their membranes. Our results complement with the design strategy by showing differential antibacterial activity, though range bound in membrane lysis (Fig. 2).

AS04 is very active against Gentamicin resistant MRSA ATCC 33592 and *E. coli* MG1655. AS06 was comparatively less effective against both the species. AS01, AS02, AS03 and AS08 are showing different activity levels against Gram-positive and Gram-negative species (Fig. 2). AS02 was very effective against *M. smegmatis* ATCC 607, a model organism for tuberculosis. Hemolytic activity of AS01 was slightly higher compared to other test peptides, which are well within the acceptable threshold (Table 1).

**Figure 3:**
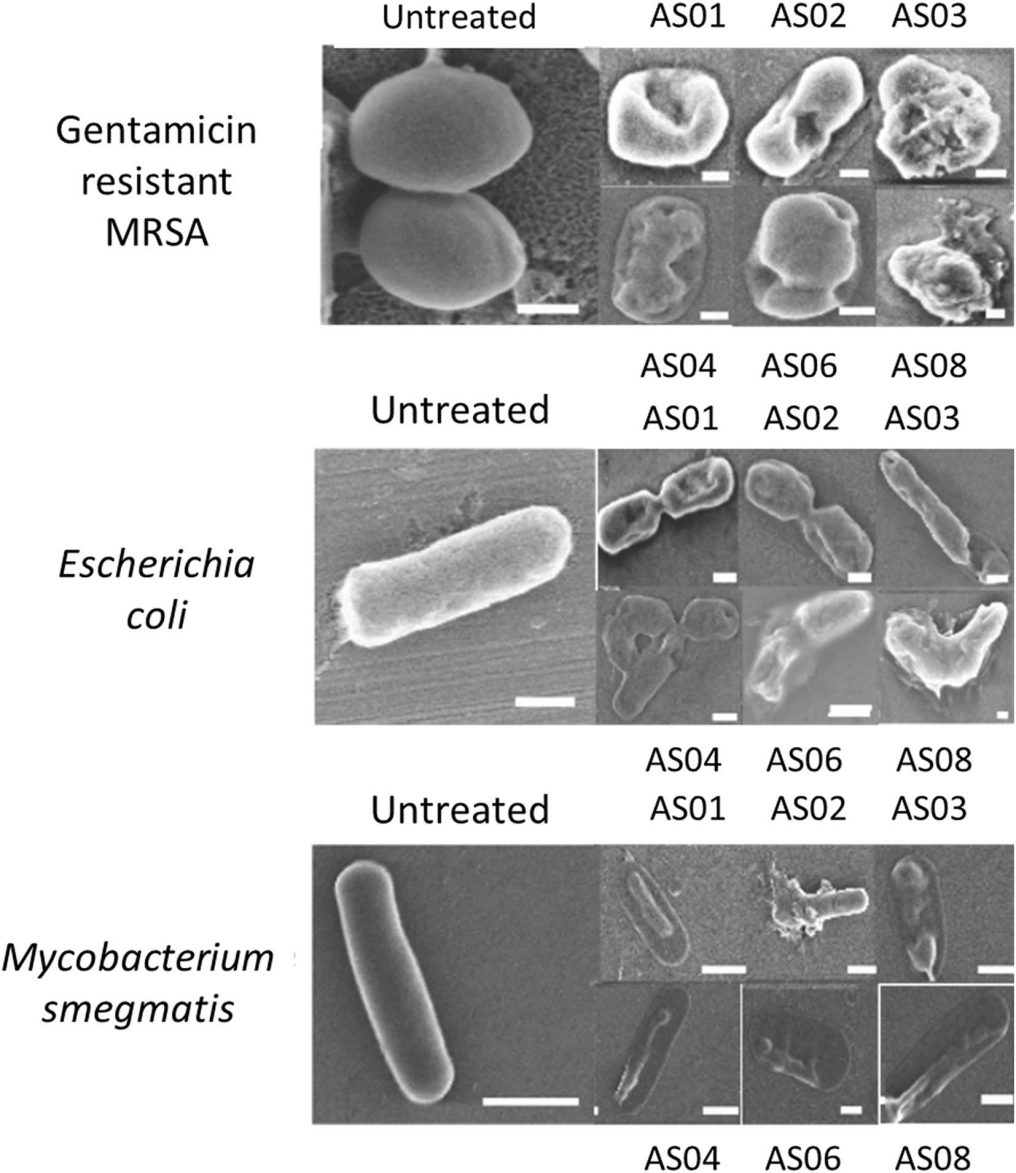
FESEM images of bacterial membranes with designed peptides. FE-SEM images of peptides against Gentamicin resistant MRSA (ATCC 33592), *Escherichia coli* (MG1655) and *M. smegmatis*. All the scale bar shown is 200 nm in length, except that of *M.smegmatis* is shown as 500 nm in length.

Field Emission Scanning Electron Microscopy (FE-SEM) was performed to make a qualitative assessment of bacterial membrane disruption by the designed peptides (Fig. 3). FESEM images of treated samples compared to the untreated ones provide a qualitative picture of the antimicrobial peptide activity on bacterial membranes.

The antibacterial activity of the peptides was also tested in the presence of 50 percent serum. The serum is a representative bio-fluid comprised of multiple factors responsible for reduced therapeutic activity under in vivo conditions^15^. Insertion of D-enantiomers within a peptide sequence has been reported to address issues like enzymatic degradation, ion sensitivity and mammalian cytotoxicity^16^.

The hetero-chiral peptide sequences were shown to retain their antibacterial activity in serum, indicating their stability and retention of therapeutic potential in physiological conditions. All the peptides were showing very impressive activity profile in micro-molar range (Fig. 2, Table 1). However, the peptide AS06 has not been very effective.

### Activity of peptides against resistant clinical isolates

Antibacterial assay (in vitro) against model bacterial membrane suggests that AS01, AS02, AS03, AS04, and AS08 are significantly active against Gentamicin resistant MRSA ATCC 33592, *E. coli* MG1655 and *M. smegmatis* ATCC 607. Among these five peptides AS01 and AS08 have shown hemolytic activity against human RBC. The other three peptides are therefore selected in the next step of evaluating their antibacterial activity against clinical isolates.

**Figure 4:**
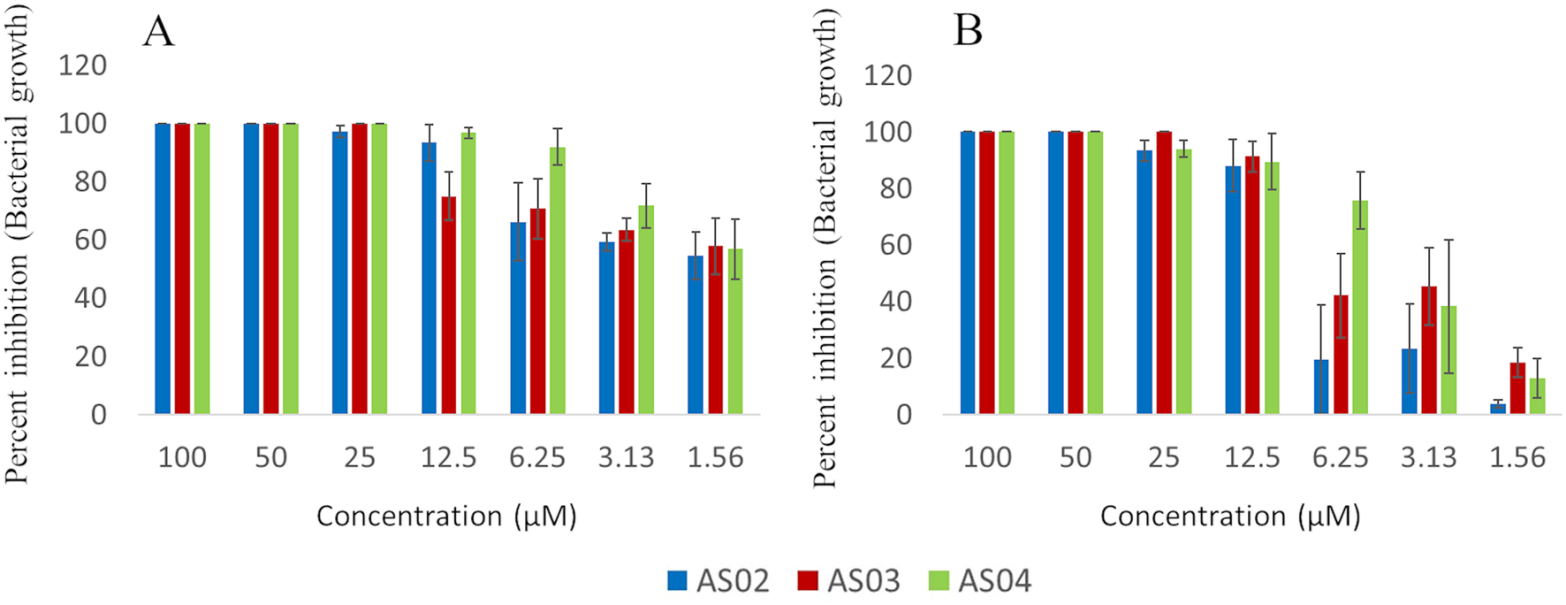
Antimicrobial activity of the peptides AS02, AS03 and, AS04 against clinical isolates of multi-drug resistant (**A**) *Escherichia coli* and (**B**) drug resistant *Staphylococcus aureus*. The error bars represent standard deviation with in the graphs.

Primary screening of the antibacterial activity of the peptides was performed against clinical isolates obtained from a tertiary referral hospital (Details in supplementary information, section 3). Resistant *Staphylococcus aureus* (Gram-positive) and *E.coli* (Gram negative) are the two clinical bacterial strains assayed in this study. The results of the antimicrobial assay are summarized in Fig. 4 and supplementary Table 2. All the peptides were active against Gram-positive and Gram-negative bacteria with MIC values ranging from of 25–50 μM.

### Molecular dynamics simulations

**Figure 5:**
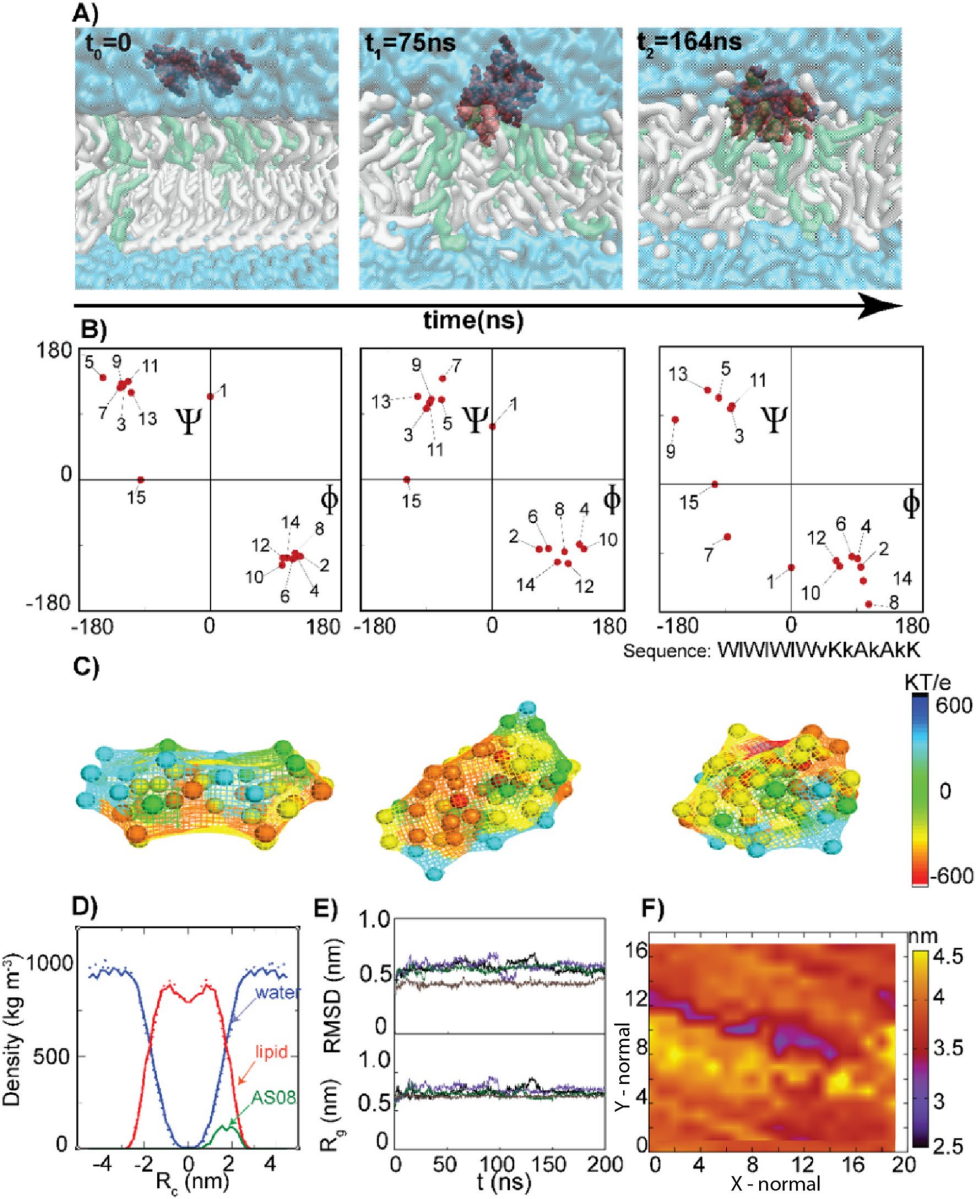
Interaction of the antimicrobial peptide AS08 with POPC:POPG (3:1) membrane. (**A**) Insertion of AS08 peptide as a function of time. (POPC: white, Quick surf format, POPG: green, Quick surf format, AS08 Peptides: red, VdW format, Water: cyan, Quick surf format). (**A**) Three time points t_0_, t_1_ and t_2_ represents the mean structure of the largest clusters formed at three different events; (1) while the peptide is in water medium, (2) while the AS08 peptide interacts with the lipid bilayer and (3) time of penetration of the peptide into the upper membrane. Plotted using open source software VMD (https://www.ks.uiuc.edu/Research/vmd/) (**B**) Ramachandran plot for AS08 peptide at various time frames (t_0_, t_1_ and t_2_). Red dots represent Ramachandran φ, ψ combinations of individual amino acids of AS08 peptide at respective times of analysis t_0_, t_1_ and t_2_ (**C**) Electrostatic potential maps of AS08 peptide assembly at t_0_, t_1_ and t_2_, plotted using MATLAB tool (**D**) Mass Density of the lipid membrane system showing difference between AS08 free system (dotted lines) and the system after peptide interaction for 200 ns (full lines). (**E**) RMSD (Upper panel) and Radius of Gyration Rg (Lower panel) of the peptides during the course of simulation. (**F**) Bilayer thickness analysis of the system after 200 ns of MD simulation showing areas of membrane thinning (blue and red represents thinner areas and yellow thicker areas). MD simulations and analysis were performed using GROMACS suite.

Based on the design philosophy and in-vitro assay results, we have chosen AS03, AS05, AS06 and AS08 as model peptides for MD simulation. AS05 has been excluded from antibacterial assays post synthesis, for not having the desired purity, but included in MD simulations. AS08 is a longer 15 mer sequence compared to the other 12 mer peptide. AS02 is very effective against all the three species, where as AS05 and AS06 are comparably less effective ones. The candidates for MD simulations are chosen by keeping this variety in their activity as a bench mark (Fig. 5, Supplementary Figures 4, 5,6).

To understand the mode of action and possibly support future designs on syndiotactic polypeptide backbones, while fine-tuning local electrostatics in modulating antimicrobial activity and cellular toxicity, we performed Molecular Dynamics (MD) simulations of four representative model systems for 200 ns each. It is difficult to fully elucidate and comparatively assess the membrane interaction mechanism of all the four peptides AS03, AS05, AS06, and AS08, across bacterial model membranes in realistic time scales. But the early events of peptide-membrane interactions and possible modes of disruption can be mapped. We used earlier reported bilayer model membranes composed of POPC and POPG in the ratio of 3:1^25^.

The peptides were chosen such that they are significantly different in their respective electrostatic fingerprints, topology, and extent of antimicrobial potential. We setup four independent simulations, each initially composed of four peptides randomly placed above the POPC: POPG bilayer. In all the four experiments, individual peptide units undergo aggregation forming an assembly within the first 10 ns of the simulation, followed by adsorption of the aggregated peptide complex to the upper membrane. The peptide complex established contacts mostly through the positively charged amino acid residues. The contacts between peptides and membrane were established through positively charged amino-acids especially lysine of the peptide complex, binding to the phosphate groups of POPC and POPG, at the water–membrane interface. Membrane interaction of AS peptides started after 20 ns, facilitating the insertion of the peptide complex into the upper membrane.

The contacts of the peptide complex to the upper membrane were very stable for AS03 and AS08, while AS05 and AS06 were showing signs of deeper penetration, though no appreciable penetration has occurred within our simulation time (200 ns). At the end of the MD simulation, half of the peptide complex has penetrated well inside the upper membrane, though longer timescales would have resulted in further penetration. In conclusion, the driving force facilitating penetration of the antimicrobial peptides AS03, AS05, AS06 and AS08 in the POPC: POPG lipid bilayer during MD, was provided by electrostatic attractions exerted by the phosphate groups localized at the regions of the bilayer on the external side of the membrane. The observation of these events at the interface crossing points suggests that the multimeric assembly is crucial to the antimicrobial activity of the peptide (Fig. 5, Supplementary Figures 4–7), a finding, which may be useful in future designs. We calculated the partial density profiles along with the membrane normal for the POPC: POPG bilayer, water and the peptide. Both without and with the peptide, the POPC: POPG membrane displayed similar density profiles ranging from − 3 to 3 nm (bilayer center at 0 nm) with a lipid head-group maximum at − 1.1 and 1.1 nm. The peptide density profiles were seen to be ranging between 1 and 3 nm, for AS03, AS05, AS06 and AS08 peptides (Fig. 5, Supplementary Figures 4–7).

We observe that peptides with lower MIC values and therefore better antimicrobial potencies like AS08 and AS03 tend to remain closer to the outer membrane, whereas AS05 and AS06 penetrate further into the membrane (Fig. 5, Supplementary Figures 4–7). The results indicate an interesting observation that peptides with larger values of electrostatic potential gradient across the structure may result in having them (peptides) being more adhered to the outer layer of the membrane and probably with more lytic abilities.

This observation is further corroborated from the heat map generated from bilayer thickness analysis (Fig. 5F, Supplementary Figure 4F, 5F and 6F). The gramicidin helical structure was found to be more or less retained in all the four peptides, an observation that can be deduced from Ramachandran Map (Phi-psi combinations), combined with radius of gyration and root mean square deviation profiles (Fig. 5, Supplementary Figures 4–7).

## Discussion and conclusion

All eight molecular models were distinctly different in their electrostatic fingerprints, achieved by sequential design variations, conserving the basic amphipathic character. The difference in electrostatic fingerprints was found to have translated to their antibacterial properties as well. Molecular dynamics simulations also endorse specificity in their respective membrane interactions of all the four model peptides tested.

The key questions asked while designing this work were (1) explore the possibility of designing a new genre of bio-active materials beyond isotactic stereochemistry by making systematic variations in polypeptide stereochemistry; (2) generating a series of novel antimicrobial agents that are either as effective or better than reported peptides antibiotics; (3) maintain similar activity levels even in serum conditions, which was the principal drawback of peptide drugs (4) generation of few short polypeptide sequences that can be further evaluated in clinical trials. We were successful in meeting all the objectives, especially in conserving the same level of antimicrobial activity in serum. The antimicrobial potential of the peptide sequences was retained in the presence of serum, indicating their stability and retention of therapeutic potential in physiological conditions. At least 5 out of 6 sequences are showing exceptional broad-spectrum activity, especially against gram negative and Mycobacterium species, with comparable or better MIC values than peptide drugs reported so far^21^. We have further tested the antibacterial activity of these peptides against clinical isolates. The antimicrobial activities of the peptides were less in resistant clinical isolates, compared to model bacterial strains.

Several modes of action of AMP’s have been proposed^35^, and the contentious issue primarily is whether the peptide is targeting the membrane, followed by its disruption or it penetrates the membrane invading cytoplasm, affecting cellular metabolism. Earlier MD simulation studies have suggested that AMP induced membrane perturbation is a stochastic event and this uncertainty makes it hard for a given bacterial species to develop resistance^35^. However, positive charge, hydrophobicity and amphipathicity were found to be the principal factors that influence AMP-membrane action. The anionic lipopolysaccharide and peptidoglycan enhance membrane binding, followed by clear segregation of polar and apolar functional groups in amino-acid side-chains to opposite regions of the peptide structure. To achieve this, our designs should primarily conserve the designed backbone architecture irrespective of its binding environment, so that the amphipathicity designed by optimizing the sequence with cationic and hydrophobic residues will be meaningful. This extra stability was provided by the harmonious local and non-local electrostatic interactions operative in a peptide backbone, an observation discussed in one of our earlier reports^36^. The LDLD backbone stereochemistry has favorable local electrostatics due to NH and CO bonds with opposite polarity coming close in a syndiotactic beta helix (Fig. 1), unlike isotactic alpha helical folds^37^, a phenomenon described in detail elsewhere^38,39^. Our results clearly suggest that peptides initially undergo aggregation forming a single globular unit, before interacting with the membrane, conserving the beta helical conformation throughout the simulation. The preferential binding of the cationic sidechain regions to the outer leaflet of the membrane and hydrophobic regions to the inner leaflet is a consequence of conformational stability of the peptide and asymmetry in bilayer compositions. Two peptides with relatively larger cationicity or larger values of electrostatic potential gradient therefore remain more on the surface, while other two tend to penetrate deep. Our wet lab experiments suggest that surface bound peptides AS03 and AS08, have MIC values in the range 1–10 µM, whereas AS06 has MIC value close to 50 µM. Bilayer thickness analysis (Fig. 5) suggest that AS08 peptide is the one that maximally disrupt the membrane among the four peptides tested. The two important design clues from MD simulations may be summarized as follows; (1) Association of individual peptide units precede membrane interaction, an observation more close to the mechanism proposed in barrel stave and toroidal pore models and (2) Optimal balance of charge and hydrophobicity is the key to their activity; with relatively more positively charged peptides remain more surface bound and therefore more effective in lysing the membrane.

In this work, we have designed eight model systems, synthesized and functionally characterized six of them. Four of the designed peptides were chosen for Molecular Dynamics simulations in search of design clues detrimental in antimicrobial activity, though we had minimal interest in performing a mechanistic investigation. Two are having high antimicrobial activity and the other two are poor performers. We re-created their membrane interaction in a Molecular Dynamics simulation using GROMOS force field, taking inspiration from Richard Feynman statement of ‘What I cannot make, I do not understand’^40^. The principal objective of this work, hinging around the application of developing ‘peptide-based antibiotics’, was to explore the possibility of creating conformationally stable stereo-chemically diverse short polypeptide constructs, that can conserve the designed amphipathicity while interacting with the membranes. The antimicrobial potential of the peptide sequences with syndiotactic stereochemistry has been retained in the presence of serum, suggesting their activity under physiological conditions. This will help in fine tuning future designs of interacting peptide molecules as potential leads, specific to membrane types and disease conditions.

## Supplementary Information

Supplementary Information.

Supplementary Video S1.

Supplementary Video S2.
