# Supplementary material for: Antimicrobial effects of syndiotactic polypeptides
Source: Sci Rep. 2021 Jan 19;11:1823. doi: 10.1038/s41598-021-81394-2 (PMC7815786; doi:10.1038/s41598-021-81394-2)
Supplement: Supplementary file 1 — Supplementary Information. [file 41598_2021_81394_MOESM1_ESM.docx]

**Antimicrobial Effects of Syndiotactic Polypeptides**

Prakash Kishore Hazam ^1, 2^ ^#^, Chimanjita Phukan^3^, Akhil R ^1^.^#^, Anjali Singh ^1#^, and Vibin Ramakrishnan ^1^*

1. Molecular Informatics & Design Laboratory, Biosciences & Bioengineering, Indian Institute of Technology Guwahati, Assam - 781039, India

2. Department of Pharmacology &Toxicology, National Institute of Pharmaceutical Education and Research Guwahati, Assam – 781125, India

3. Guwahati Medical College Hospital, Bhangagarh, Guwahati, Assam – 781032, India

Email: vibin@iitg.ac.in

# All the authors have equally contributed

**Supplementary Information**

**Contents**

| **Section** | **Details** | **Page no** |
| --- | --- | --- |
| 1 | Electrostatic potential map | 2 |
| 2 | Primary Characterization of the synthesized peptide | 3 |
| 3 | Antimicrobial activity and bacteria resistance profile | 4 - 5 |
| 3 | Molecular dynamics simulation | 6-10 |
| 5 | Legends for Supplementary Video files | 11 |

**Section 1: Electrostatic potential map**


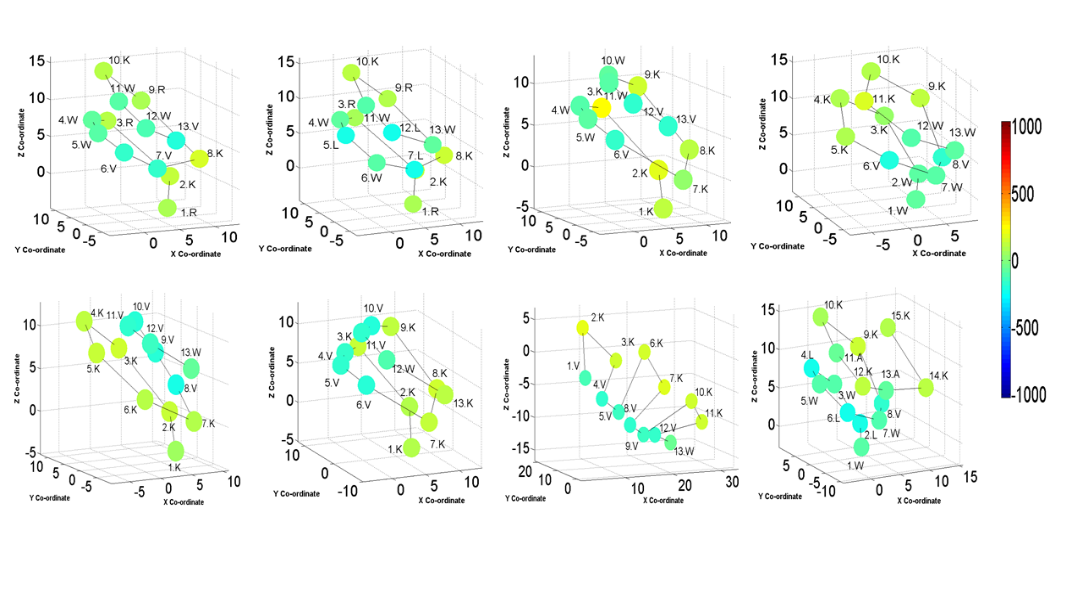


**Supplementary Figure 1. E**lectrostatic potential distribution profiles of peptides AS01 – AS08. The electrostatic potential is expressed in KT/e unit.

**Section 2: Primary Characterization of the synthesized peptide.**


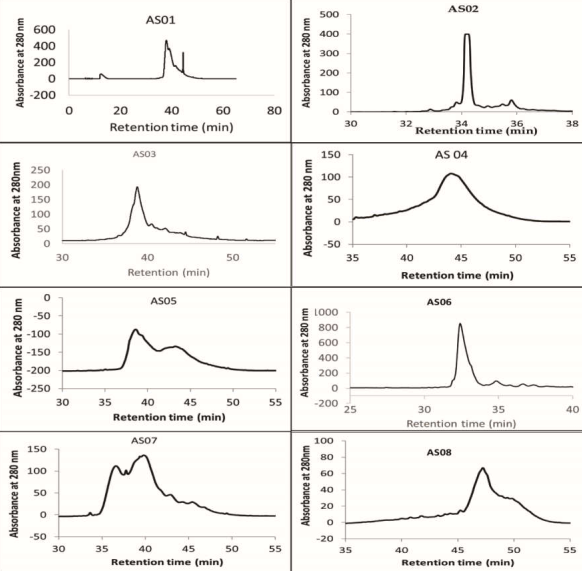


**Supplementary Figure 2:** HPLC profile of the synthesized peptides of AS series (AS01 – AS08)

**Supplementary Table 1.** The observed (from MALDI experiment) and expected mass of the peptides AS01 – AS08

| S No | Peptide code | Expected Mass (Da) | Observed Mass (Da) |
| --- | --- | --- | --- |
| 1 | AS01 | 1911.126 | 1912.542 |
| 2 | AS02 | 1953.173 | 1954.756 |
| 3 | AS03 | 1827.103 | 1828.324 |
| 4 | AS04 | 1914.19 | 1916.291 |
| 5 | AS05 | 1596.086 | 1596.110 |
| 6 | AS06 | 1596.086 | 1956.76 |
| 7 | AS07 | 1566.075 | 1567.502 |
| 8 | AS08 | 1983.187 | 1983.15 |

**Section 3. Antimicrobial activity and bacteria resistance profile**

**
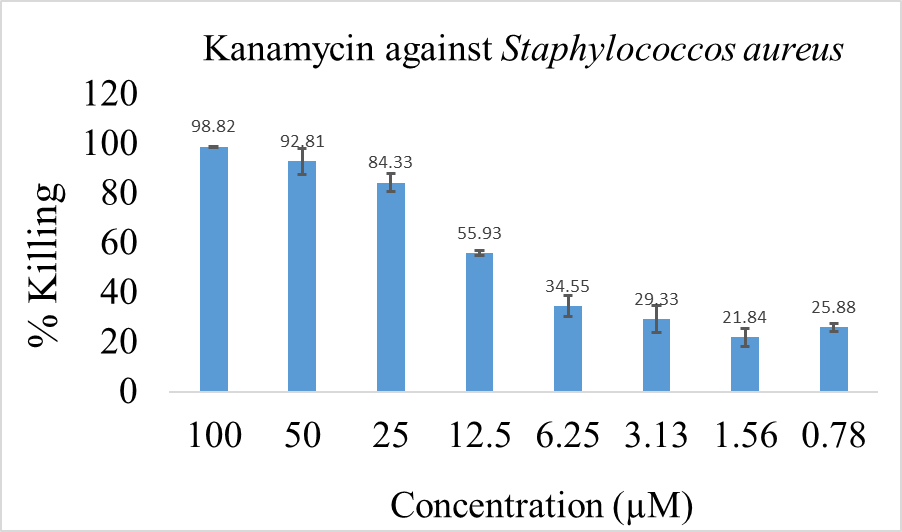
**

**Supplementary Figure 3:** Antimicrobial activity of Kanamycin against *Staphylococcus aureus* performed under identical conditions.

**Supplementary Table 2.** The *in-vitro* antimicrobial activity of designed peptides against clinical isolates of multi-drug resistant *Staphylococcus aureus* and *Escherichia coli*. The concentration is expressed in µM.

| Peptide code | *E.coli* (µM) | *S. aureus* (µM) |
| --- | --- | --- |
| AS02 | 50 | 50 |
| AS03 | 25 | 25 |
| AS04 | 25 | 50 |

**Supplementary Table 3.** Resistant *Staphylococcus aureus* and *Escherichia coli* strains used in the assay, and details of their respective resistance data (list of marketed antibiotic with which they are resistant to).

| ***Staphylococcus aureus*** | | ***Escherichia coli*** | |
| --- | --- | --- | --- |
| **Resistant antibiotic** | **Generation** | **Resistant antibiotic** | **Generation** |
| Benzyl penicillin | First-generation penicillins | Cefuroxime | Second-generation cephalosporins |
|  |  | Cefuroxin Axetil | Second-generation cephalosporins |
|  |  | Ceftriaxone | Third-generation cephalosporins |
|  |  | Ciprofloxacin | Second-generation quinolones |
|  |  | Trimethoprim/Sulphamethaoxazole | Not applicable |
|  |  | Ampicillin | Third-generation penicillins |

Hospital: Guwahati Medical College Hospital (GMC-H), Guwahati, India

**Sample Reference Numbers:**

*S. aureus*: 28401

*E.coli*: 28162

**Section 4: Molecular dynamics simulation**

**Supplementary Table 4.** Details of molecular systems studied by Molecular Dynamics Simulations. The peptide code, its corresponding sequence, number of lipids, the temperature, and the simulation time are listed.

| Peptide code | AS03 | AS05 | AS06 | AS08 |
| --- | --- | --- | --- | --- |
| Peptide sequence | KkKwWvKkKwWvV | KkKkKkKvVvVvW | KkKvVvKkKvVwK | WlWlWlWvKkAkAkK |
| Lipid (POPC:POPG) | (180:60) | (180:60) | (180:60) | (180:60) |
| Temperature (K) | 303 | 303 | 303 | 303 |
| Time(ns) | 200 | 200 | 200 | 200 |

**
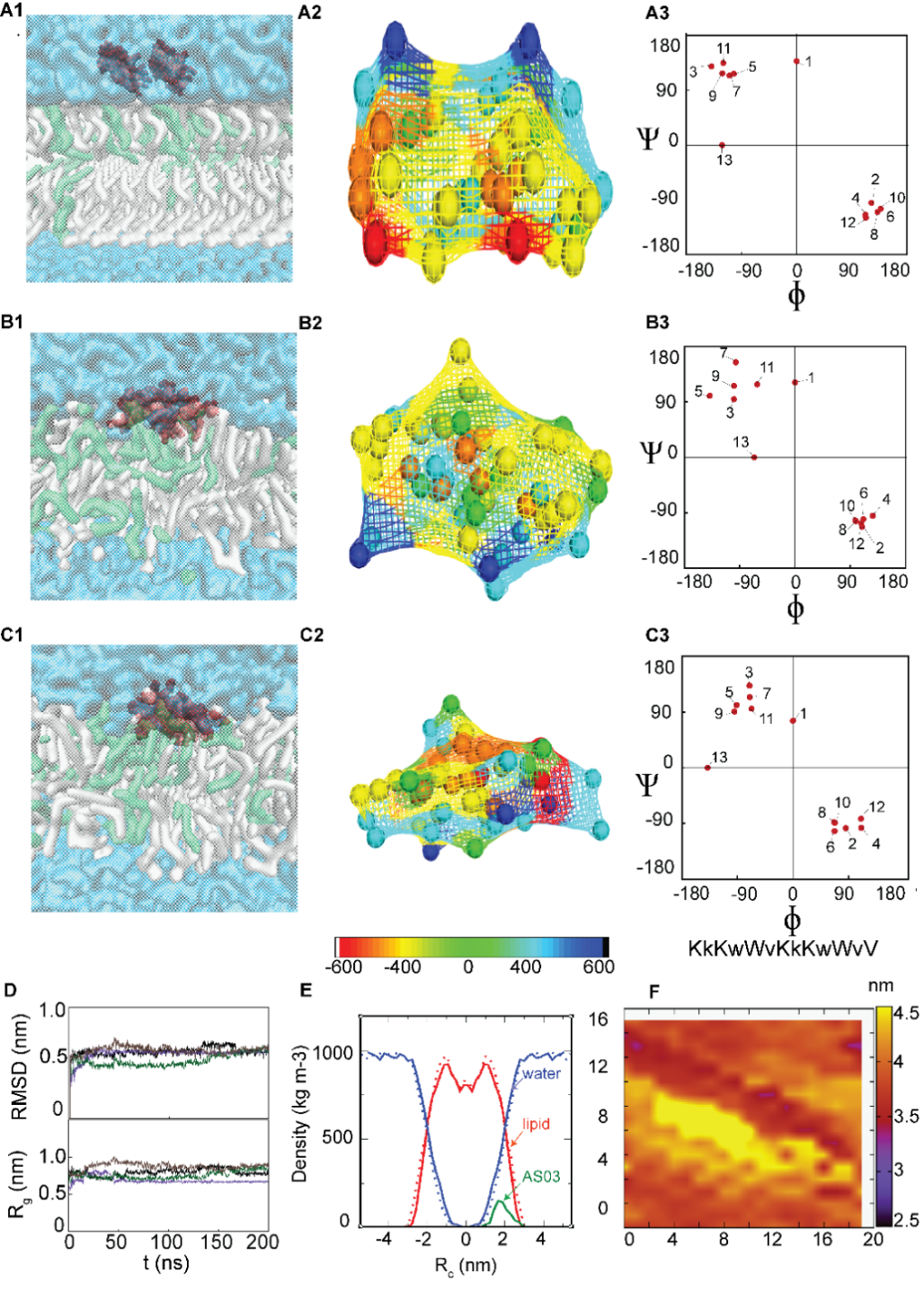
**

**Supplementary Figure 4**. Interaction of the antimicrobial peptide AS03 with POPC:POPG (3:1) membrane. (A1, B1 and C1) Insertion of AS03 peptide as a function of time. (POPC: white, Quick surf format, POPG: green, Quick surf format, AS03 Peptides: red, VdW format, Water: cyan, Quick surf format). Three time points t_0_, t_1_ and t_2_ represents the mean structure of the largest clusters formed at three different events; 1) while the peptide is in water medium, ii) while the AS03 peptide interacts with the lipid bilayer and iii) time of penetration of the peptide into the upper membrane. (A2, B2 and C2) Electrostatic potential maps of AS03 peptide assembly at t_0_, t_1_ and t_2_. (A3, B3 and C3) Ramachandran plot for AS03 peptide at various time frames (t_0_, t_1_ and t_2_). Red dots represent Ramachandran φ, ψ combinations of individual amino acids of AS03 peptide at respective times of analysis t_0_, t_1_ and t_2_. (D) RMSD (Upper panel) and Radius of Gyration Rg (Lower panel) of the peptides during the course of simulation. (E) Mass Density of the lipid membrane system showing difference between AS03 free system (dotted lines) and the system after peptide interaction for 200ns (full lines). (F) Bilayer thickness analysis of the system after 200ns of MD simulation showing areas of membrane thinning (blue and red represents thinner areas and yellow thicker areas).

**
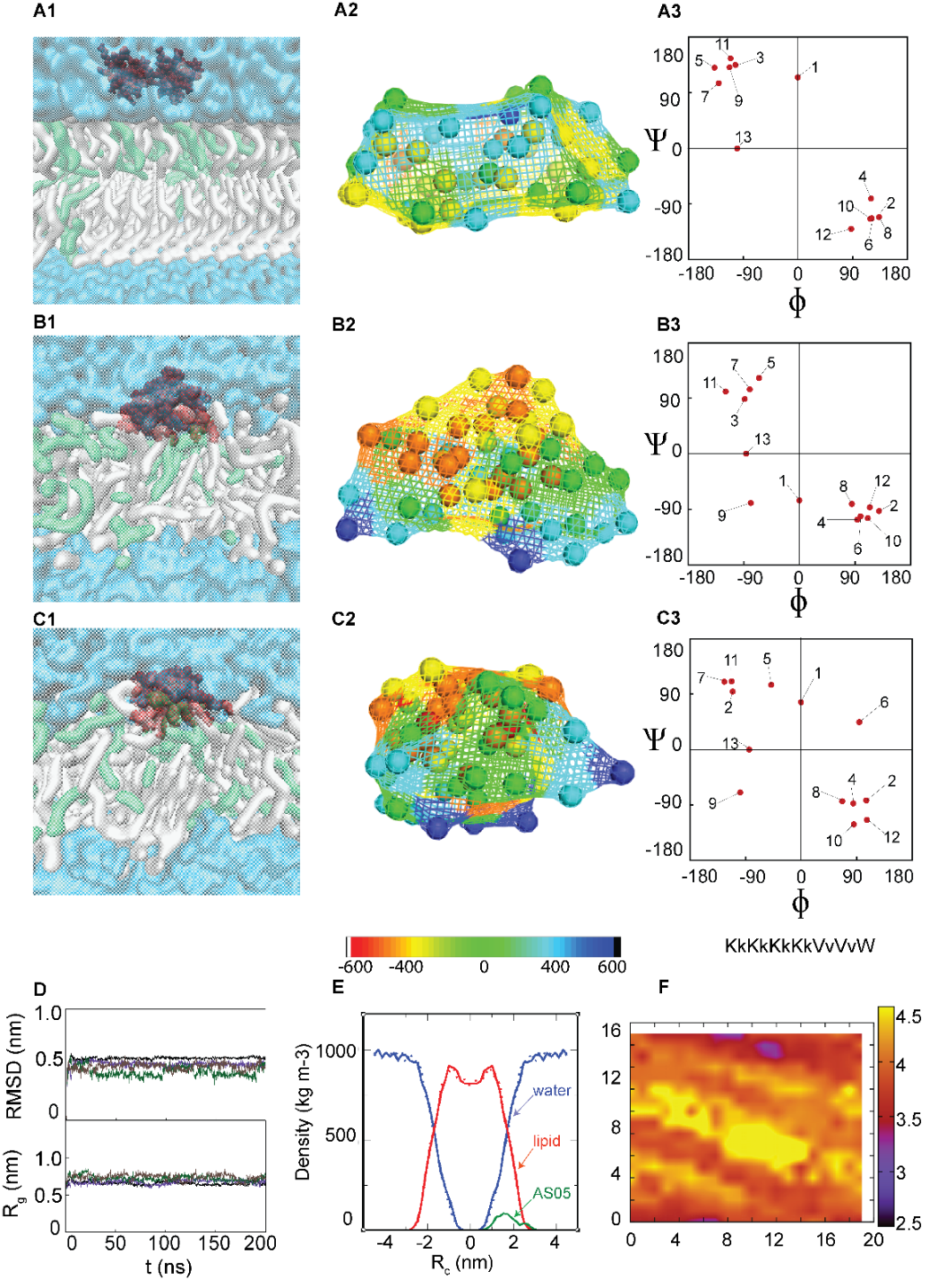
**

**Supplementary Figure 5**. Interaction of the antimicrobial peptide AS05 with POPC:POPG (3:1) membrane. (A1, B1 and C1) Insertion of AS05 peptide as a function of time. (POPC: white, Quick surf format, POPG: green, Quick surf format, AS05 Peptides: red, VdW format, Water: cyan, Quick surf format). Three time points t_0_, t_1_ and t_2_ represents the mean structure of the largest clusters formed at three different events; 1) while the peptide is in water medium, ii) while the AS05 peptide interacts with the lipid bilayer and iii) time of penetration of the peptide into the upper membrane. (A2, B2 and C2) Electrostatic potential maps of AS05 peptide assembly at t_0_, t_1_ and t_2_. (A3, B3 and C3) Ramachandran plot for AS05 peptide at various time frames (t_0_, t_1_ and t_2_). Red dots represent Ramachandran φ, ψ combinations of individual amino acids of AS05 peptide at respective times of analysis t_0_, t_1_ and t_2_. (D) RMSD (Upper panel) and Radius of Gyration Rg (Lower panel) of the peptides during the course of simulation. (E) Mass Density of the lipid membrane system showing difference between AS05 free system (dotted lines) and the system after peptide interaction for 200ns (full lines). (F) Bilayer thickness analysis of the system after 200ns of MD simulation showing areas of membrane thinning (blue and red represents thinner areas and yellow thicker areas).

**
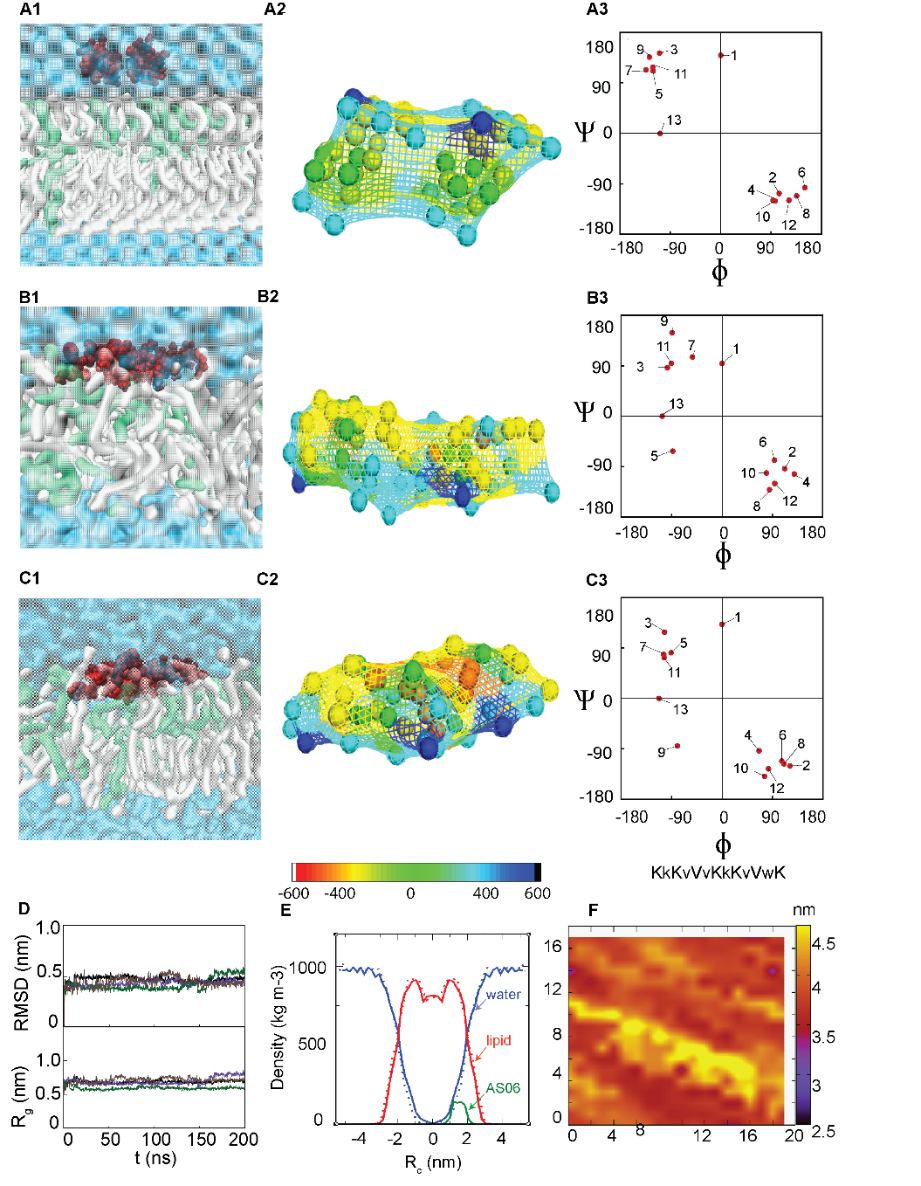
**

**Supplementary Figure 6.** Interaction of the antimicrobial peptide AS06 with POPC:POPG (3:1) membrane. (A1, B1 and C1) Insertion of AS06 peptide as a function of time. (POPC: white, Quick surf format, POPG: green, Quick surf format, AS06 Peptides: red, VdW format, Water: cyan, Quick surf format). Three time points t_0_, t_1_ and t_2_ represents the mean structure of the largest clusters formed at three different events; 1) while the peptide is in water medium, ii) while the AS06 peptide interacts with the lipid bilayer and iii) time of penetration of the peptide into the upper membrane. (A2, B2 and C2) Electrostatic potential maps of AS06 peptide assembly at t_0_, t_1_ and t_2_. (A3, B3 and C3) Ramachandran plot for AS06 peptide at various time frames (t_0_, t_1_ and t_2_). Red dots represent Ramachandran φ, ψ combinations of individual amino acids of AS06 peptide at respective times of analysis t_0_, t_1_ and t_2_. (D) RMSD (Upper panel) and Radius of Gyration Rg (Lower panel) of the peptides during the course of simulation. (E) Mass Density of the lipid membrane system showing difference between AS06 free system (dotted lines) and the system after peptide interaction for 200ns (full lines). (F) Bilayer thickness analysis of the system after 200ns of MD simulation showing areas of membrane thinning (blue and red represents thinner areas and yellow thicker areas).


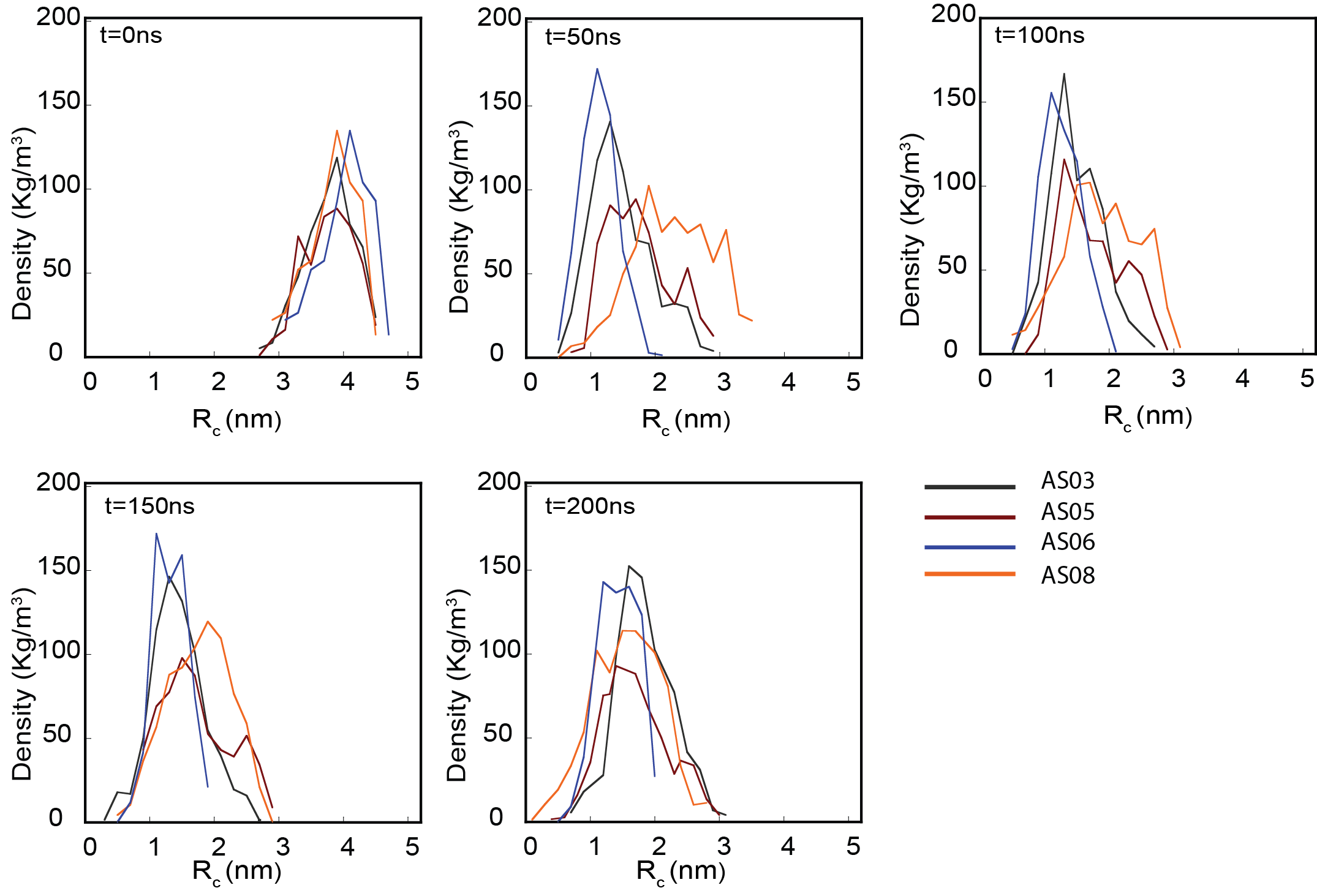


**Supplementary Figure 7**. Comparison of Mass densities of AS peptides (AS03, AS05, AS06 and AS08) at various time frames (t= 0ns,50ns, 100ns,150ns and 200ns) of MD simulations; where R_c_ is ‘relative position from the center of the bilayer in nm.

**Section 5: Legends for Supplementary video files**

**Supplementary movie** 1 (0 to 10 ns): This movie is extracted from the initial 10 ns of molecular dynamics simulations of AS08 peptide. Simulation video between 0 to 10 ns simulation clearly shows the aggregation of individual peptide units forming an assembly before membrane interaction (POPC: white, Quick surf format, POPG: green, Quick surf format, AS08 Peptides: VdW format, positively charged amino acids are shown in blue, other amino acids in red, water molecules are not shown).

**Supplementary movie 2** (10 to 100 ns): This movie displays the adsorption of aggregated peptide complex to the upper membrane followed by its insertion. (POPC: white, Quick surf format, POPG: green, Quick surf format, AS08 Peptides: VdW format, positively charged amino acids are shown in blue, other amino acids in red, water molecules are not shown).
